# The effect of qigong for pulmonary function and quality of life in patients with covid-19: A protocol for systematic review and meta-analysis

**DOI:** 10.1097/MD.0000000000022041

**Published:** 2020-09-18

**Authors:** Jing Peng, Zhimin Wu, Hongling Zhong, Yaying Zhou, Li Wang, Yu Wang, Wei Luo, Ya Liu, Linglin Zhang

**Affiliations:** a Department of Outpatient; b Department of Gynaecology and Obstetrics; c Department of Anesthesiology; d Department of Neurology, The First Hospital Affiliated Tо AMU (Southwest Hospital); e Department of Respiratory and Critical Care Medicine, Chongqing Jiangbei Hospital of Traditional Chinese Medicine; f Department of Dermatology, Chongqing Hospital of Traditional Chinese Medicine, Chongqing, China.

**Keywords:** COVID-19, pulmonary function, qigong, schemes, systematic review

## Abstract

**Background:**

Qigong is a traditional Chinese exercise method for health care, keeping fit and getting rid of diseases. It has the advantages of simple operation and few side effects. Corona Virus Disease 2019 (COVID-19) is an acute respiratory infectious disease caused by severe acute respiratory syndrome coronavirus 2(SARS-COV-2). Its clinical manifestations mainly include fever, fatigue, and dry cough. Clinical practice showed that Qigong had some therapeutic effects on pulmonary dysfunction caused by novel Coronavirus, but there was lacking in evidence of evidence-based medicine. The purpose of this protocol is to systematically evaluate the effects of Qigong on lung function and quality of life in COVID-19 patients, and to add evidence to evidence-based medicine for the clinical application of Qigong therapy.

**Methods:**

Use computer to retrieve English database (PubMed, Embase, Web of Science, the Cochrane Library) and Chinese database (China Knowledge Network (CNKI), Wanfang Database, VIP Information Chinese Journal Service Platform (VIP), Chinese Biomedical Database). In addition, we manually retrieve randomized controlled clinical research from Baidu academic and Google academic from its establishment to July 2020. Two researchers independently extracted and evaluated the quality of the data included in the study, using RevMan5.3 to do meta-analyses of articles included, without language restrictions.

**Results:**

This research evaluated the effectiveness and safety of Qigongs influence on patients pulmonary function and life quality by index such as 6-minute walk distance (6MWD), Forced expiratory volume in 1 second (FEV1), Forced vital capacity (FVC), Forced expiratory volume in 1 second/Forced vital capacity (FEV1/FVC), Forced expiratory volume in 1 second/prediction (FEV1/PRE), Self-rating anxiety scale (SAS), etc.

**Conclusions:**

This study will provide reliable evidence-based evidence for the clinical application of Qigong in the treatment of COVID-19.

**PROSPERO Registration number:**

CRD42020191877.

## 1 Introduction

COVID-19 first appeared in Wuhan, China, in early 2019,^[1]^ then broke out in China in the following months and spread around the world. Novel Coronavirus is the pathogen of COVID-19. The main sources of infection are patients and asymptomatic carriers infected with the virus. It is transmitted through droplets and direct contact. Moreover, the virus is highly contagious and people are generally susceptible to it.^[2]^ Patients are mainly characterized by fever, dry cough, and fatigue. A few patients are accompanied by symptoms such as nasal congestion, sore throat and diarrhea. Patients who are severely and critically ill may have dyspnea, acute respiratory distress syndrome, or even multiple organ failure etc. The prognosis for the elderly and those with chronic underlying diseases is poor.^[3]^ Previous studies showed that coronaviruses can replicate in large numbers in the body, with massive epithelial cell necrosis and cytokine releasing, setting off an inflammatory cascade.^[4]^ Studies have found that the high expression of cytokine is detected in the novel Coronavirus patients this time.^[5]^ The accumulation of various immune cells, cytokines and mucus in the lung tissue may obstruct gas exchange, lead to the death of a large number of alveolar cells, and seriously break the pulmonary gas exchange.^[6]^

Qigong originates from the theory of health preservation and fitness in ancient Chinese medicine and is a kind of health preservation methods. According to traditional medicine, Qigong is a mind-body exercise skill that combines body regulation, breath regulation and mind regulation.^[7]^ Long-term adherence to Qigong can effectively improve body function, promote secretion and metabolism, and enhance muscle strength.^[8]^ In this outbreak, Qigong combined with traditional Chinese and western medicine treatment of COVID-19 patients in China achieved good results.^[9]^ Qigong is easy to operate, safe, and effective, with few side effects. It has great advantages in clinical adjuvant therapy and daily health care.

Although many clinical studies have shown that Qigong assisted treatment of COVID-19 has significant effects, high cure rate, low recurrence rate, and few adverse reactions, the number of clinical trials is small, and there are differences in study design and efficacy, which affects the promotion of this therapy to some extent. Therefore, in this study, we conducted a meta-analysis to investigate the effects of Qigong on respiratory function, quality of life, and psychological status of COVID-19 patients, providing reliable evidence-based evidence for Qigong adjuvant therapy for COVID-19.

## 2 Methods

### 2.1 Protocol register

This protocol of systematic review and meta-analysis has been drafted under the guidance of the preferred reporting items for systematic reviews and meta-analyses (PRISMA). Moreover, the protocol and registration information are available at http://www.crd.york.ac.uk/PROSPERO/ (registration number: CRD42020191877.).

### 2.2 Ethics

Since this is a protocol with no patient recruitment and personal information collection, the approval of the ethics committee is not required.

### 2.3 Eligibility criteria

#### 2.3.1 Types of studies

We collected all available randomized controlled trails (RCTs) on Qigong treatment for COVID-19, regardless of blinding, publication status, region, but language will be restricted to Chinese and English.

#### 2.3.2 Research objects

1. With a definite diagnosis of COVID-19;
2. Mini-mental state examination (MMSE) score is 21;
3. No chronic obstructive pulmonary disease (COPD) or any other respiratory disease.

#### 2.3.3 Intervention measures

Treatment group: Qigong was combined with other similar energy practices, such as yoga techniques, and meditation. Control group: The control group only received conventional therapy such as routine health guidance and/or drug treatments.

#### 2.3.4 Outcome indicators

1. Main outcome: ① 6-minute walk distance (6MWD); ② Forced expiratory volume in 1 second (FEV1).
2. Secondary outcomes: ① Forced vital capacity (FVC); ② Forced expiratory volume in 1 second/Forced vital capacity (FEV1/FVC); ③ Forced expiratory volume in 1 second/prediction (FEV1/PRE); ④ Self-rating anxiety scale (SAS); ⑤ Self-rating depression scale (SDS); ⑥ Various quality of life scales.

### 2.4 Exclusion criteria

1. Study published repeatedly;
2. Study whose literature is abstract and conference papers, in which the original data cannot be obtained;
3. Study whose data is incomplete or where there are obvious errors that cannot be handled after contacting the author;
4. Study with wrong random method;
5. Moderate or severe heart disease (Grade III or IV, New York Heart Association);

### 2.5 Retrieval strategy

“COVID-19”, “Qigong”, “Gongfa”, “conduction exercise”, “Tai Chi” were searched in Chinese databases, including CNKI, Wanfang Data Knowledge Service Platform, VIP, and China Biomedical Database. English retrieval words such as “ COVID-19”, “ Qigong ”, “ Gongfa ” and “ Tai Chi ”, etc. were searched in English database, including PubMed, EMBASE, Web of Science, the Cochrane Library. In addition, manual search was conducted on Baidu academic and Google academic. The retrieval time was from the establishment of the database to July 2020, and all domestic and foreign literatures on Qigong treatment for COVID-19 were collected. Taking PubMed as an example, the retrieval strategy is shown in Table 1.

**Table 1:**
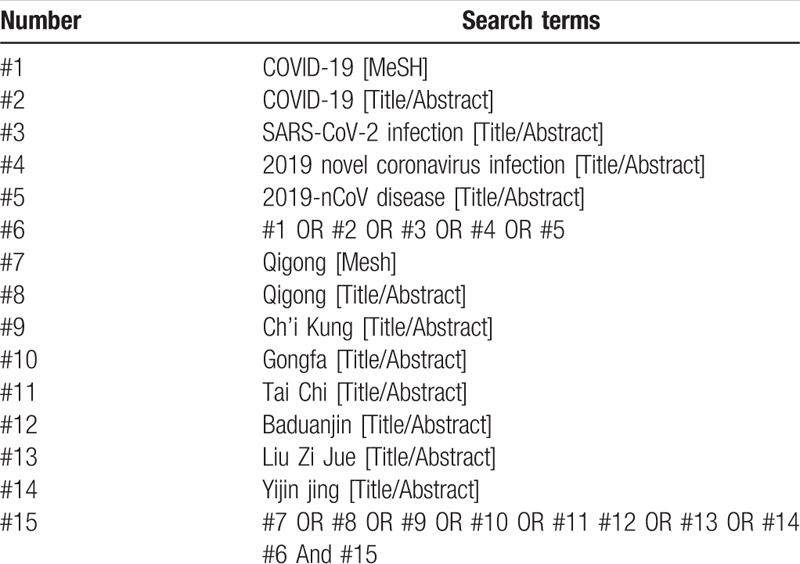
Search strategy in PubMed database.

### 2.6 Data screening and extraction

**Figure 1:**
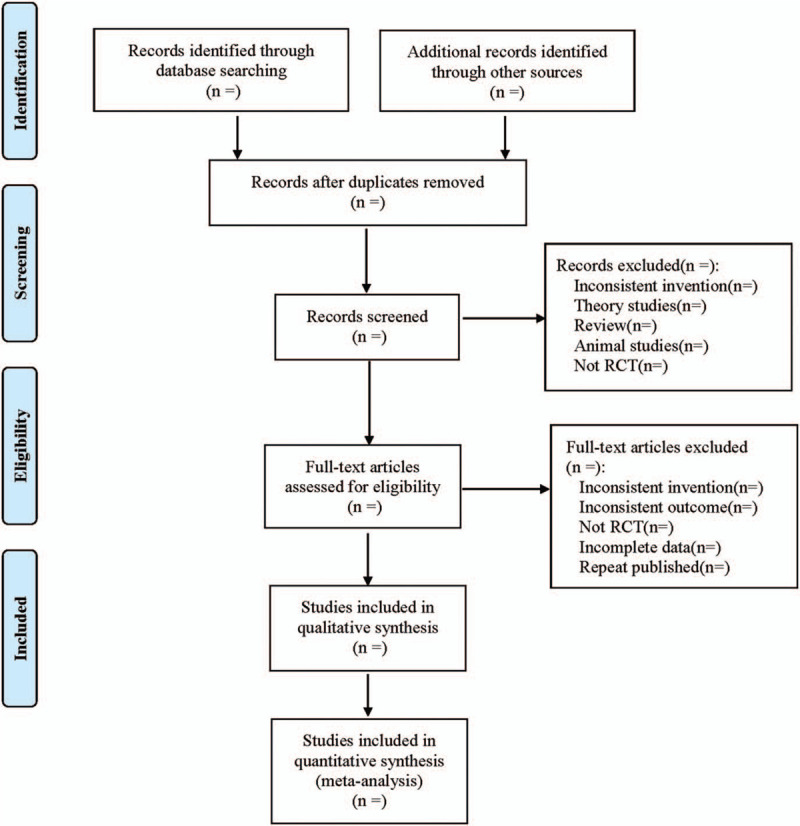
Flow diagram.

Cochrane Collaboration System Reviewer Manual Version 5.0 was used as a reference for the method of selection in the study. According to the PRISMA flow chart, EndNote X^7^ document management software was utilized by 2 researchers to independently screen the documents based on the above inclusion and exclusion criteria before mutual check. Those difficult to determine whether included in the study, would be discussed and judged with a third researcher. At the same time, Excel 2013 was used to extract relevant information, including: ① Clinical features (title, first author, publication year and month, sample size, sex ratio, average age, average course of disease); ② Intervention measures: the name, action points, training frequency and course of Qigong used in the treatment group, as well as other therapies used in the treatment group and their frequency and course of treatment; other treatment measures used in the control group, such as drug name, administration method, frequency, course of treatment, etc.; ③ Evaluation factors of risk bias in randomized controlled studies; ④ Observation indicators. The literature screening process is shown in Figure 1.

### 2.7 Literature quality evaluation

Built-in Risk bias evaluation tool of Review Manager 5.3 Software (the Cochrane collaborations tool for assessing risk of bias) was used to assess the risk bias in the included studies. Two researchers determined the literatures from 3 levels, including low-risk, unclear, and high-risk based on the performance of the included literature in the above evaluation items. After completion, they would recheck. In case of a disagreement, they would discuss. If no agreement could be reached, a decision would be made in consultation with researchers from the third party.

### 2.8 Statistical analysis

#### 2.8.1 Data analysis and processing

The RevMan 5.3 software provided by the Cochrane Collaboration was used for statistical analysis. ① For dichotomous variables, relative risk (RR) was used for statistics. For continuous variables, weighted mean difference (WMD) was selected when the tools and units of measurement indicators are the same, standardized mean difference (SMD) was selected with different tools or units of measurement, and all the above were represented by effect value and 95% confidence interval (CI). ② Heterogeneity test: Q test was used to qualitatively determine inter-study heterogeneity. If *P* ≥ .1, there was no inter-study heterogeneity, if *P* < .1, it indicated inter-study heterogeneity. At the same time, *I*^2^ value was used to quantitatively evaluate the inter-study heterogeneity. If *I*^*2*^ ≤ 50%, the heterogeneity was considered to be good, and the fixed-effect model was adopted. If *I*^*2*^ > 50%, it was considered to have significant heterogeneity, the source of heterogeneity would be explored through subgroup analysis or sensitivity analysis. If there was no obvious clinical or methodological heterogeneity, it would be considered as statistical heterogeneity, and the random-effect model would be used for analysis. Descriptive analysis was used if there was significant clinical heterogeneity between the 2 groups and subgroup analysis was not available.

#### 2.8.2 Dealing with missing data

If data is missing or incomplete, we will contact the corresponding author to obtain the missing data. If not, this study will be removed.

#### 2.8.3 Subgroup analysis

Do respectively subgroup analysis according to the treatment group of Qigong assisted with other traditional Chinese medicine therapies and Qigong alone; According to the age of the patients, they can be divided into 4 subgroups: minors, young people, middle-aged people, and elderly people for subgroup analysis. According to the stage of COVID-19, it can be divided into 4 subgroups: initial, middle, severe, and convalescent; subgroup analysis was carried out according to the types of Qigong used; subgroup analysis was performed according to the course of treatment.

#### 2.8.4 Sensitivity analysis

In order to test the stability of meta-analysis results of outcomes, a one-by-one elimination method will be adopted for sensitivity analysis.

#### 2.8.5 Assessment of reporting biases

For the major outcome indicators, if the included study was ≥10, funnel plot was used to qualitatively detect publication bias. Eggers and Beggs test are used to quantitatively assess potential publication bias.

#### 2.8.6 Evidence quality evaluation

The Grading of Recommendations Assessment, Development, and Evaluation (GRADE) will be used to assess the quality of evidence. It contains 5 domains (bias risk, consistency, directness, precision, and publication bias). And the quality of evidence will be rated as high, moderate, low, and very low.

## 3 Discussion

In the respect of traditional Chinese medicine, this novel Coronavirus belongs to the category of “plague (Wenyi)”, and its main nature of disease is noxious dampness, which can also be called noxious dampness plague. The location of disease is the lung and spleen, and its basic pathogenesis is “dampness, toxin, stasis and closure”. The main clinical symptoms are recessive fever, cough, fatigue, poor appetite, thick greasy tongue coating.^[10]^ Plague is recorded as “plague” in the “*General Treatise on the Causes and Syndromes of Diseases (Zhubing Yuanhou Lun)*”, and it can be also called “Yi li”, “Yi Qi” and “Yi Bing” etc.^[11]^

Qigong is one of the 5 treatment methods in ancient Chinese medicine. It has a long history, but in the process of development, it was endowed with religious significance and lost its scientific value. Today, the research of Qigong in Traditional Chinese medicine is making solid progress in the direction of normalization, scientification, and internationalization.^[12]^ It is mentioned in the “ *Plain Questions (Suwen)*” that diseases cannot attack the body if the normal level of immunity is maintained. Qigong focuses on strengthening the human immunity to resist diseases, which can improve the human bodys resistance to pathogenic microorganisms as well as its ability to adjust and adapt to them.^[13]^ A large number of clinical trials have shown that Qigong can enhance immune function, reduce the risk of infection, improve the prognosis, improve sport endurance, quality of life, and the activity of daily living.^[14]^ Qigong can strengthen exercise of the chest and abdomen muscle, increase the depth of breathing, relieve dyspnea,^[15]^ and improve lung function.^[16,17]^ It can also relieve psychological stress, depression and anxiety, and improve sleep quality.^[18,19]^ The difference between Qigong and ordinary sports lies in that Qigong enriches the unique “mind regulation” based on the “body regulation ” and “breath regulation ” in sports,^[20]^ which is the core of Qigong. The balance of qi, blood, Yin and Yang can be adjusted by regulating the spirit and emotions, so as to achieve a healthy state of “ correspondence between human body and natural environment (Tian-ren Xiangying)”.^[21]^ There are different Qigong exercises such as Tai Chi, Ba Duan Jin, Liu Zi Jue, and Yi Jin Jing, etc., which should be selected clinically for patients to treat diseases, and the Qigong with high physical exertion should not be selected for weak patients.

At present, many trials on Qigong treatment for COVID-19 have been widely reported, but systematic and correct evaluation is lacking. Therefore, it is necessary to objectively evaluate the impact of Qigong on COVID-19 patients by evidence-based medicine, promote Qigong treatment, and provide scientific and evidence-based exercise prescription for clinic. However, this study also has some limitations. Different basic treatments will have a great impact on the results. Some severe patients cannot complete Qigong exercise, and there is a lack of large sample size and high-quality randomized controlled trials. At the same time, due to the limitation of linguistic competence, only English and Chinese literature were searched, and studies in other languages may be ignored, which may lead to certain publication bias.

## Author contributions

**Data collection**: Jing Peng and Linglin Zhang.

**Funding support**: Li Wang and Linglin Zhang.

**Literature retrieval**: Zhimin Wu and Hongling Zhong.

**Software operating**: Yaying Zhou and Yu Wang.

**Supervision**: Wei Luo and Ya Liu.

**Writing – original draft**: Jing Peng and Linglin Zhang.

**Writing – review & editing**: Jing Peng and Linglin Zhang.

## Abbreviations

Abbreviations: 6MWD = 6-minute walk distance, CI = confidence interval, CNKI = China Knowledge Network, COPD = chronic obstructive pulmonary disease, COVID-19 = Corona Virus Disease 2019, DM = diaphragm muscle mobility, FEV1 = Forced expiratory volume in one second, FEV1/FVC = Forced expiratory volume in one second/Forced vital capacity, FEV1/PRE = Forced expiratory volume in one second/prediction, FVC = Forced vital capacity, GRADE = The Grading of Recommendations Assessment, Development, and Evaluation, MMSE = mini-mental state examination, PRISMA = the preferred reporting items for systematic reviews and meta-analyses, RCTs = randomized controlled trails, RR = relative risk, SARS-COV-2 = severe acute respiratory syndrome coronavirus 2, SAS = self-rating anxiety scale, SDS = self-rating depression scale, SMD = standardized mean difference, VIP = VIP Information Chinese Journal Service Platform, WMD = weighted mean difference.
